# An Age-Structured Approach to Modelling Behavioural Variation Maintained by Life-History Trade-Offs

**DOI:** 10.1371/journal.pone.0084774

**Published:** 2014-01-08

**Authors:** Matthew H. T. Chan, Peter S. Kim

**Affiliations:** School of Mathematics and Statistics, University of Sydney, Sydney, New South Wales, Australia; Vrije Universiteit, Netherlands

## Abstract

There have been numerous empirical studies on the fitness consequences of behavioural syndromes in various animal taxa; however, the ecological and evolutionary implications on a population level are still poorly understood. To better understand these implications, we develop a non-linear age-structured mathematical model to qualitatively examine the evolutionary consequences of a heritable boldness personality trait within an animal population. We assume that this heritable boldness trait is positively correlated with boldness towards predators and intraspecific aggressiveness. This assumption leads to a growth/reproductive success versus mortality trade-off, which is thoroughly investigated and documented in the literature. Another life-history trade-off we include in the model is future versus current reproduction, which was shown by Wolf et al. [1] to be a possible mechanism for the evolution of behavioural syndromes within an animal population. The stability of the system is analysed, whereby the characteristic equation is in the form of a homogeneous Fredholm equation of the second kind which depends on both the perturbation and equilibrium solution. The system is found to be stable due to the competition between individuals of similar boldness acting as a negative feedback mechanism. Using numerical simulations we examine the qualitative features of the solution to the system. In particular, we investigate the interplay between the mutation and competition strength between two individuals with different boldness, whereby we find that an increasing competition range acts to push individuals to both extremes of the shy-bold axis, while an increasing mutation range counteracts this effect. This qualitative trait of aggregation of individuals around the shy and bold extremes is also found when examining different birth, mortality and competition functions.

## Introduction

Recent studies suggest that species across a wide range of taxa exhibit behavioural syndromes (also known as animal personality), that is, correlated suites of behaviours across different situations which are also stable across time. This is despite the fact that a high degree of behavioural plasticity would theoretically allow for an individual to maximize their fitness in different situations and environments. Many studies give evidence that behavioural types within a behavioural syndrome are moderately heritable and stable over the entire life of the individual, thereby being a target of natural selection due to their influence on life history strategy (for a review on heritability, see Oers et al. [2]) [3]. For example, Dingemanse et al. [4] found that exploratory behaviour for wild Great Tits (*Parus Major*) was both repeatable and heritable, and unrelated to age, body condition or sex. This was found to influence important life-history traits such as offspring production, recruitment rates and survival, which implies that personality in Great Tits is selected upon, leading to evolutionary change. In addition to heritability, there is strong support that phenotypic correlations between components of personality often originate from strong underlying genetic correlations. A well documented example is the strong correlation between boldness towards predators, high activity level and intraspecific aggressiveness observed in various animal taxa [5]–[8].

A fundamental behavioural type is the shy-bold continuum, where boldness is defined to be the propensity for an individual to take risks [9]–[12]. This behavioural type has been documented in over 60 species across multiple taxa, suggesting its importance as a component of animal personality, particularly due to empirical studies often finding that boldness is consistent across multiple contexts (but see Sinn & Moltschaniwskyj [13]). Studies give evidence that an individual’s position on the shy-bold axis often correlates with crucial life-history traits such as reproduction and survival, due to its correlation with related traits such as antipredator behaviour, mate choice, intraspecific aggression, growth, body mass, foraging behaviour, exploration/dispersal behaviour, recruitment and social behaviour [14], [15].

Moreover, crucial behavioural types such as shy-boldness influence and reflect important life-history trade-offs, such as growth versus mortality, mortality versus reproductive success and current reproduction versus future reproduction. For example, in stickleback fish populations, individuals that are more aggressive towards conspecifics are also bolder towards predators. While a high level of intraspecific aggressiveness is instrumental in gaining resources (mates, food etc.), the correlated antipredator boldness may incur an increased mortality risk [6], [16].

Although there have been a large number of empirical studies which have investigated shy-boldness across multiple contexts and the fitness consequences in various animal taxa, there have been very few studies which examine, particularly from a mathematically qualitative perspective, the evolutionary and ecological implications that shy-bold personality variation has on a population. Examples include life history and demography, dispersal and invasion speeds, speciation and disease spread (see Wolf & Weissing [17] for a detailed review on such implications). However, to understand the implications there must first be a framework on the more fundamental question of how shy-bold variation is maintained within a population and how it evolves over time. There are a few hypotheses in the literature which attempt to answer this; for example, a density- and frequency-dependent selection model or a similar model which includes phenotypic plasticity (Wilson et al. [18]) or a life-history trade-off model (Wolf et al. [1], Reale et al. [19]). In this study, we focus on the latter, in particular a trade-off between current and future reproductive success as proposed by Wolf et al. [1]. This postulates that an asset protection principle drives the evolution of personality variation; individuals with high future reproductive expectations are more risk-averse than individuals who have low future reproductive expectations. Using an agent-based model, Wolf et al. [1] showed that a dimorphism in an inheritable exploration trait, whereby individuals who explore the environment thoroughly invest in future reproduction/fitness and individuals who explore the environment superficially invest in current reproduction/fitness, results in a polymorphism in traits, such as antipredator boldness and intraspecific aggressiveness.

We formulate a continuous age- and boldness-structured model based on two life-history trade-offs: current versus future reproduction as proposed by Wolf et al. [1], and also mortality versus growth/reproductive success, which is due to the positive effects of intraspecific aggressiveness and the negative effects of antipredator boldness. Instead of assuming a heritable exploration trait as in the study by Wolf et al. [1], we assume that shy-boldness is heritable and is directly correlated with exploratory behaviour, in line with the commonly identified exploratory-boldness behavioural syndrome in empirical studies [9], [20]. Thus, shy individuals are thorough explorers who invest in future reproduction and suffer low risk of mortality, while bold individuals are superficial explorers who invest in current reproduction, but incur a greater mortality risk due to predation associated with inappropriate boldness.

## Methods

### Model Formulation

The boldness of an individual is given by 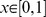, whereby 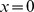 and 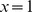 correspond to the extremes of shy and bold behaviour, respectively. We denote 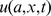 to be the frequency of individuals of age 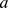 with boldness 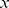 at time 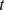 and use the McKendrick-von Foerster equation to model the age structure, given by
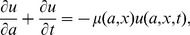
(1)with boundary condition
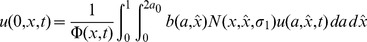
(2)and initial condition
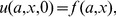
(3)where 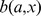 is the birth function, 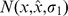 is the mutation function, 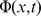 is a function governing competition between individuals, 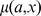 is the mortality function and 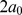 is the total fertile period of an individual. We give a specific form for each of these functions below, but also consider alternative forms for these functions in the subsequent sections to show how they can affect solutions.

We define the birth function 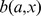 to be
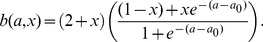
(4)


**Figure 1:**
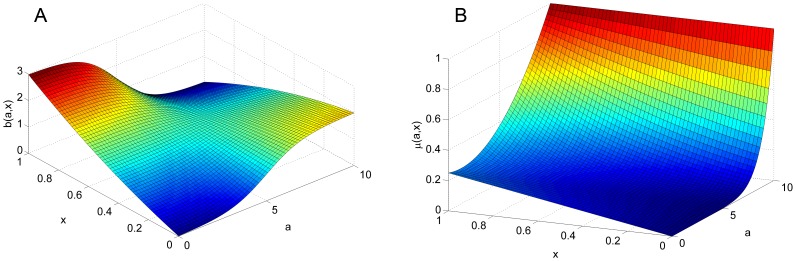
Birth and mortality functions. Panel A and B show 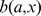 and 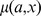 respectively. These two functions are defined such that they reflect the future versus current reproduction and growth/reproduction success versus mortality life-history trade-offs.

The factor 
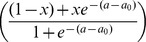
 is defined such that shy individuals reproduce towards the end of their fertile periods, while bold individuals reproduce at the start of their fertile periods. The 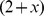 factor represents the increase in reproduction gained from a given boldness value due to aggressive behaviour when competing against conspecifics for resources (food, mates). Specifically, it increases with respect to boldness and represents the maximum number of offspring which an individual can have for a given boldness 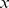. In particular, an individual with boldness 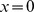 will have 2 offspring, whereas an individual with boldness 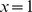 will have 3 offspring. See Figure 1 for a plot of 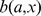.

The factor 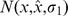 dictates mutation of the offspring, resulting in different boldness values than their parents, where 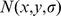 is a normal distribution with mean 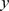 and standard deviation 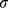, that is,
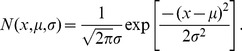
(5)


The competition experienced by an individual with boldness value 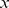 at time 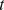, due to other individuals adopting similar strategies as a consequence of their boldness, is denoted by 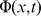. It is defined as
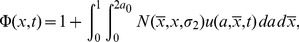
(6)where 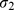 represents the strength of competition between two individuals with different boldness levels. The competition factor in the boundary condition Eq. (2) serves to reduce reproduction rates if there are too many individuals adopting similar strategies by having similar boldness values.

Finally, the mortality function 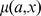 is given by
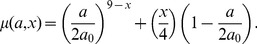
(7)


The mortality function 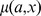 is defined such that shy individuals suffer the least mortality due to their risk-averse behaviour towards predators, in contrast to bold individuals who suffer the greatest mortality due to risk-prone behaviour towards predators. The left term accounts for an intrinsic mortality that is higher for bolder individuals and increases with respect to age due to senescence. The right term represents the penalty for a given level of boldness, where mortality is higher for bolder individuals and decreases with respect to age. This is illustrated in Figure 1. Note that 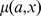 represents a mortality rate and not a probability of mortality; as such 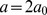 is the maximum fertile age, not the maximum life span. Thus, Eq. (7) implies that the average remaining life span after an individual’s fertility period is 1 day.

Throughout this study we set 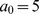, 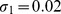, 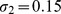 and initial condition 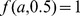, for 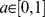, and zero everywhere else. Thus, individuals have a maximum lifespan of 10 years and begin in cohorts of age 0 to 1 with an intermediate boldness level of 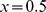.

## Results

### Stability Analysis

The solution to Eq. (1) is given by
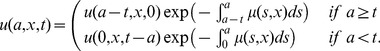
(8)


We denote the stable age and boldness distribution to be 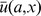, which is given by (via substituting into the McKendrick-von Foerster equation)
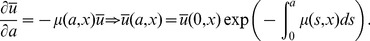
(9)


First we examine the boundary condition without the competition factor, which makes the boundary condition, and consequently the system, linear. We test stability by letting 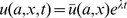. This gives
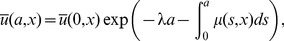
(10)and the boundary condition Eq. (2) becomes



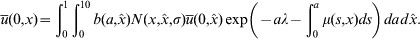
(11)By biological reasoning, the existence of the competition factor 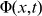 as a negative feedback must be crucial to the stability of the equilibrium 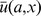. Note that the dependence on boldness 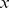, in particular mutation along the shy-bold axis, means that another approach is needed to analyse the stability than the case of classical linear age-structured models. Mathematically analysing the stability of Eq. (1) with boundary condition Eq. (11) can be achieved by employing classical Fredholm theory [21], [22]. Fredholm’s first and second theorem state that Eq. (11) has a non-trivial 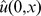 with eigenvalue 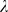 if and only if 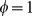 is a zero of
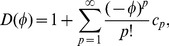
(12)where



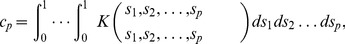
(13)

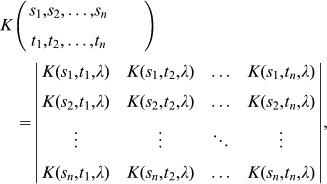
(14) and



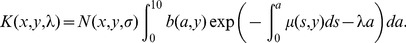
(15)Additionally, if 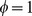 is a zero of multiplicity 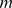 of 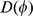, then Eq. (11) possesses at least one, and at most 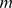, non-trivial linearly independent solutions.

The kernel 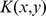 used in this model is a bounded and integrable function, which was proved to be a sufficient condition for the series in Eq. (12) to converge for all 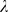 [21]. Numerically, we find that 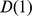 is monotonically increasing as 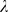 increases due to the 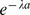 term in the integrand of Eq. (15), that is, 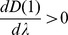 for 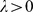, with an asymptote at 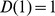. Thus, if 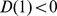 for 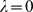, then there exists some 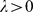 for which 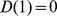. Numerically computing the first few significant terms of Eq. (12), we can show that 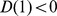 for 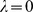 and that 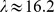 satisfies Eq. (12). This indicates that the system reaches a stable age and shy-boldness distribution which either increases or decreases exponentially (depending on the form of the birth, mortality, competition and mutation functions) when there is an absence of competition, as expected from linear age-structured population modelling theory.

For the full problem, where the competition factor is included, we first observe that the solution cannot be unbounded, regardless of the possibility of local instability, due to the following upper bound:
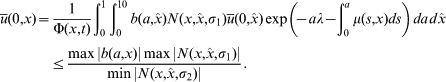
(16)


To investigate the local stability, we linearise the boundary condition by letting 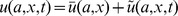, where 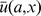 denotes the equilibrium solution and 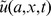 denotes a small perturbation. Substituting this into the boundary condition and linearising, we obtain
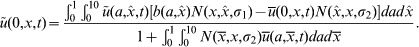
(17)


Then, we test the growth of the perturbation by setting 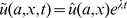, where 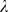 is the eigenvalue of the linear operator 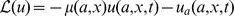 and 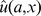 is the corresponding eigenfunction. Substituting this into Eq. (1), we find that 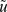 satisfies
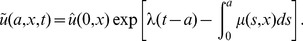
(18)


**Figure 2:**
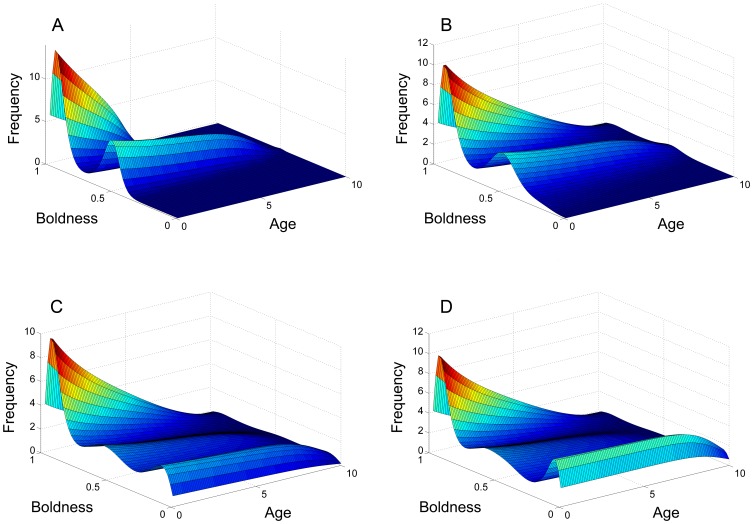
Evolution of the population over time. The system was numerically simulated using an Upwind scheme and stopped at certain 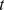 values. Panels A, B, C and D correspond to 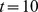, 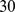, 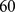 and 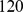 respectively. Bold individuals appear to experience the fast growth due to the future versus current reproduction trade-off.

Using Eq. (8) and Eq. (9), we obtain
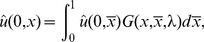
(19)where



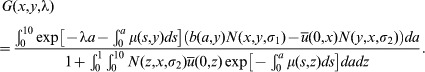
(20)Again, the eigenvalue is determined by a homogeneous Fredholm integral equation, with 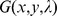 as the kernel. Note that at the trivial equilibrium 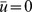, Eq. (20) reduces to the kernel for the linear case without competition Eq. (15), thus 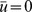 is locally unstable, as we have determined above. For the non-trivial equilibrium, we can reuse the argument for the case without the competition factor to deduce stability. However, note that explicitly calculating 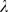 in this case is more difficult than the case without the competition factor because of the dependence on the equilibrium solution 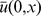 in the integral equation, for which we have no closed form. From numerical simulations, such as the one shown in Figure 2, we can see that the system has a stable non-trivial equilibrium. This is reaffirmed by numerically obtaining the long-term steady state 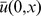 and substituting into Eq. (20) to find that 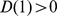 for 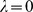, which implies that there does not exist a 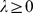 such that 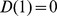. Thus the system is locally asymptotically stable.

### Simulations and Other Forms of 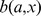 and 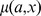


**Figure 3:**
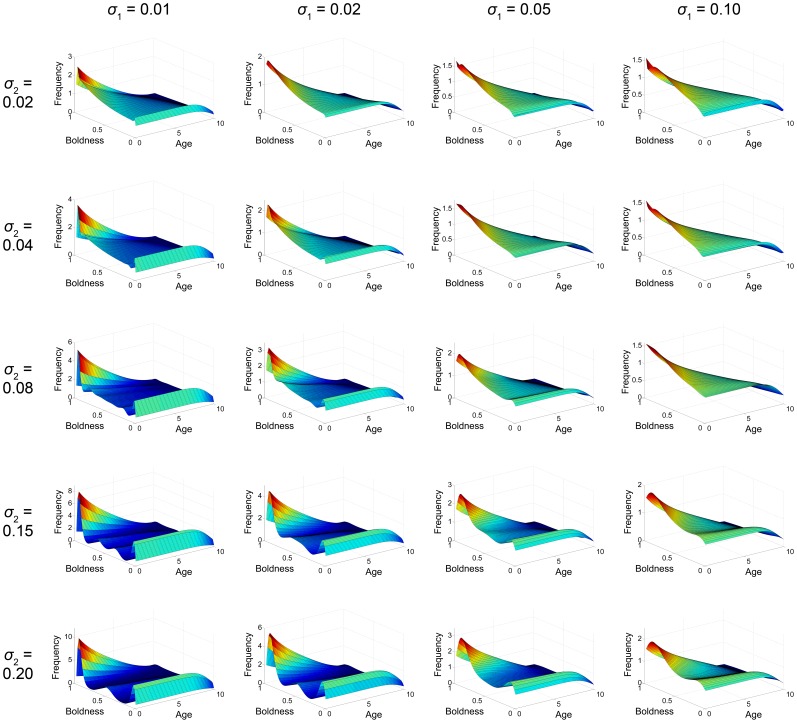
Comparison of solutions with different 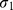 and 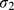. The system was numerically simulated using an Upwind scheme until the solution reached an equilibrium state. All cases reach equilibrium at approximately 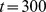, although the solution does not change substantially (minor shifts in distribution, but no big qualitative changes) after 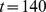. See also Figure 4.

**Figure 4:**
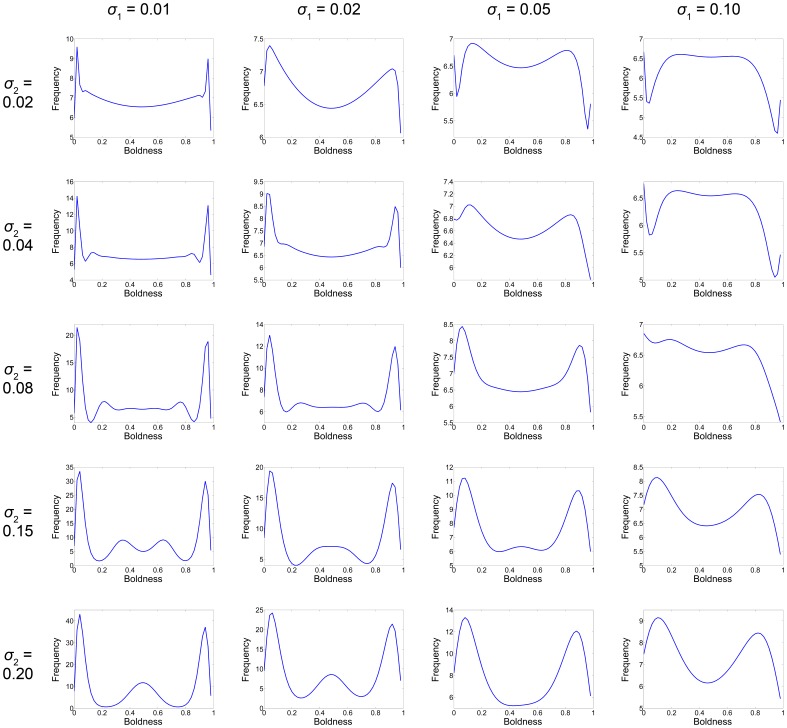
2D Comparison of solutions with different 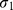 and 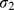. The system was numerically simulated using an Upwind scheme until the solution reached an equilibrium state and then integrated along the age axis for simplification. Hence, frequency here is a measure of the total population of a certain boldness value. See also Figure 3.

The effect of competition as a negative feedback not only induces stability in the equilibrium solution but also qualitatively affects the form of the solution, as shown in Figure 3 and Figure 4. The table of figures illustrates the relationship between mutation and competition, in particular how varying their values will alter the distribution of the population across the shy-bold continuum. Most notably, an increase of the competition range 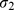 has the effect of polarising individuals to either side of the shy-bold continuum, whereas an increase in the mutation range 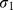 acts to counter this by spreading the population more evenly across the shy-bold axis.

**Figure 5:**
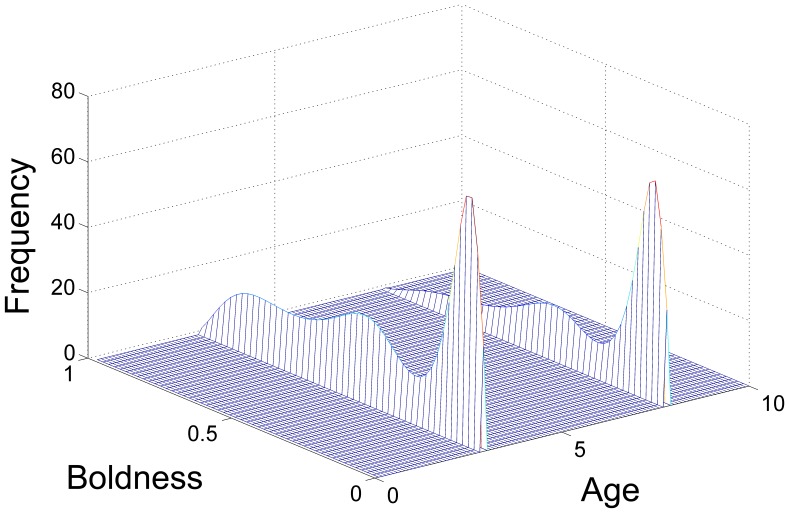
Periodic solutions of the system. The birth function was modified to 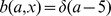 to gain a periodic solution. Individuals are clustered in cohorts, in which the shy-boldness distribution within the cohort changes over time.

Depending on the form of the birth 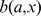 and mortality 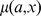 functions, it is possible for 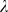 in Eq. (20) to have an imaginary part and thus render 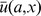 to be periodic. However, this is unlikely for smooth forms of 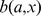 and 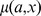, due to the requirement of Eq. (12); if 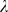 were to have a finite imaginary component, then there is the extra condition of the imaginary component of the series to converge to zero. In general, we have found that only non-smooth 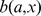 of a form similar to
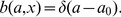
(21)allows for a periodic solution, as shown in Figure 5.

**Figure 6:**
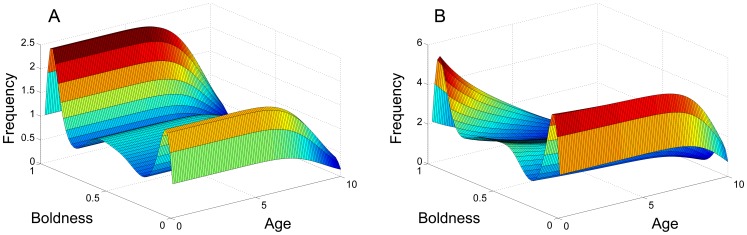
Comparing two different life-history trade-offs. Panel A and B show the case where there is only future versus current reproductive trade-off and only growth/reproductive success versus mortality trade-off respectively. Both panels are of the system at an equilibrium state.

We simulate the two cases where there is only future versus current reproduction trade-off and only growth/reproductive success versus mortality trade-off. These are shown in panel A of Figure 6 and panel B of Figure 6 respectively. The simulations indicate that either case reaches a stable equilibrium. Notably, both cases share the same qualitative feature of having isolated peaks at both shy and bold extremes.

For only future versus current reproduction, we change the birth and mortality functions to
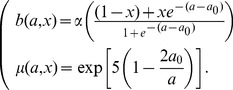
(22)


For only growth/reproductive success, we change only the birth function to
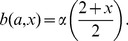
(23)


## Discussion

Studies on animal personality, in particular the shy-boldness behavioural type in animals, have observed that there are life-strategy trade-offs due to repeatability of behaviour and limited behavioural plasticity across contexts, despite the advantages of a flexible behavioural response. Many studies in the literature recognise that these trade-offs may have important ecological and evolutionary implications for an animal population, yet there have been very few mathematical studies to qualitatively examine this [23]. Using an agent-based model, where individuals participate in several games in each time step, Wolf et al. [1] demonstrated that a population dimorphism for an exploration trait (either a very thorough or superficial explorer), driven by a life-history trade-off, corresponded to a behavioural syndrome within the population, specifically, a correlation between exploration, boldness and aggressiveness. Furthermore, using a discrete mathematical model, they showed that this dimorphic population is stable over time, that is, it is protected from invasions by mutant individuals. In this study, we have presented a continuous age- and boldness-structured model with the aim of giving a qualitative insight into the evolutionary implications of a shy-boldness behavioural type maintained via a similar life-history trade-off within a population.

**Figure 7:**
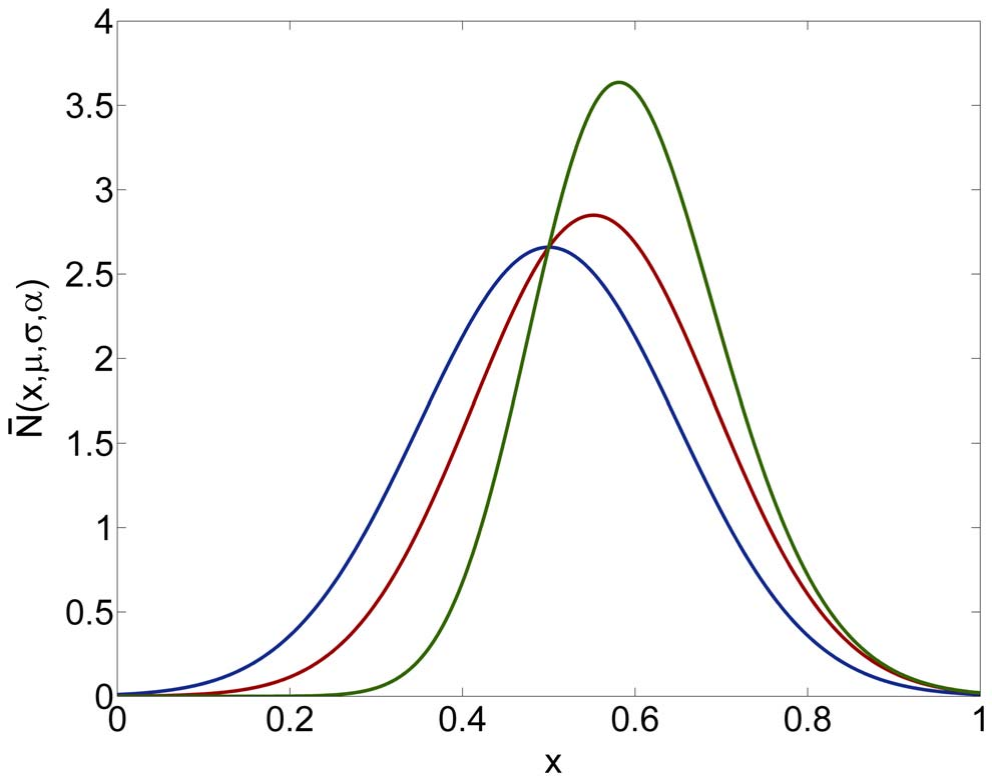
The skew normal distribution. Plots of 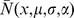, where 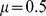, 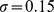 and skewness 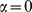, 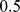, 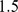 corresponds to the blue, red and green line respectively.

The functions used in this model were designed to be mathematically elegant yet roughly conforming to reality. Different forms of these functions, particularly the normal distributions used in the competition and mutation factors, could change the qualitative features of the solution dramatically. The normal distribution is perhaps a suitable representation for mutation rates in reality, and promisingly, there have been efforts to quantify the heritability of behavioural types (see Dingemanse & Reale [23] and references therein). However, competition between shy-bold individuals, assuming a boldness-aggressiveness behavioural syndrome, is not likely to be dictated by a normal distribution. Instead, it is likely to be represented by a distribution that is skewed towards bold individuals, as shy individuals, by our definition, are not as aggressive as bold individuals are. Moreover, an individual is more likely to be successful when competing against other individuals with lower boldness. Thus, a more realistic form is the skew normal distribution (shown in Figure 7), given by
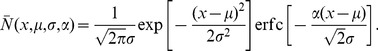
(24)


**Figure 8:**
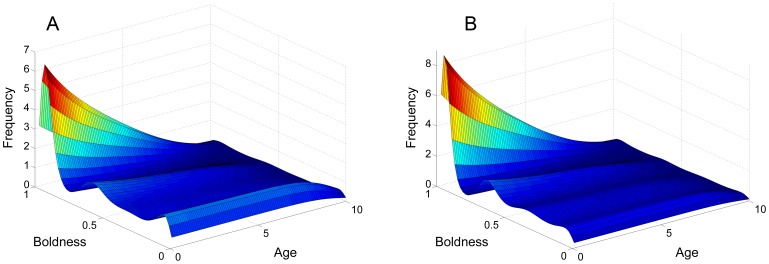
Solutions generated when competition is skewed towards bold individuals. Plots of 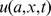 at the equilibrium state, with the skew normal distribution function 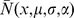 replacing the normal distribution function in the competition function Eq. (6). Panel A and B correspond to the case where 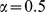 and 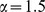 respectively.

As shown in Figure 8, this gives rise to qualitatively different solutions to those generated by the competition function using a normal distribution, since there is more incentive for individuals to be bold.

On the other hand, further assume that shy individuals are less competitive than bold individuals. This can be modelled by simply multiplying Eq. (24) by a factor of 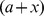, where 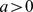 controls the difference in competition strength of shy and bold individuals. We set 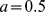, which gives a competition function of the form
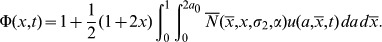
(25)


**Figure 9:**
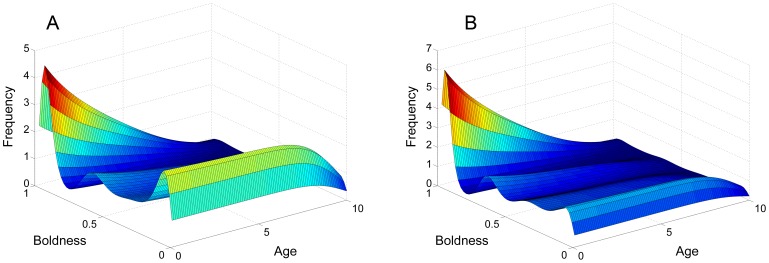
Solutions generated when competition is both skewed towards bold individuals and scaled according to boldness. Plots of 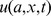 at the equilibrium state, with the modified competition function Eq. (25). Panel A and B correspond to the case where 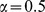 and 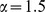 respectively.

This generates solutions where the qualitative feature of two peaks at both shy and bold extremes is more evident, as shown in Figure 9. In a similar vein, different forms of the distribution for the competition factor should be considered for different trade-offs, as different trade-offs are expected to impact competition for resources in their own way and in turn generate qualitatively different solutions.

Understanding heterogeneities in the form of behavioural syndromes in an animal population is important in predicting dispersal behaviour. In this regard, reaction-diffusion equations have long been used to model animal dispersal behaviour, mainly due to the ease of analysing such equations. However, these models assume a homogeneous population where an individual’s dispersal behaviour is an uncorrelated random walk. By the central limit theorem, this leads to a Gaussian movement distribution which results in a wavefront propagating at a constant velocity. In reality, many animals follow a leptokurtic (fat-tailed) movement distribution, which means that the wave front propagates at an accelerating rate towards a constant velocity or without bound [24], [25]. Thus many classical reaction-diffusion models severely underestimate invasion speeds of certain insects or animals. Recently, there has been much interest in correcting this discrepancy in wavespeeds by using models where a leptokurtic (fat-tailed) distribution can be adopted, such as a fractional reaction-diffusion equation or an integro-difference equation. There are a few flaws with these models: the origin of such a leptokurtic distribution is not addressed, the width of the tail strongly affects the wavespeed (this is of particular concern because data from mark-recapture studies is most scarce at the tails) and mathematical analysis is difficult on both, with most analytical results hinging on the assumption of a one-dimensional (single-species) model and linearity [26]–[28]. It has been suggested that heterogeneity within a population in the form of shy-bold variation could be the key factor behind leptokurtic movement distributions, especially since boldness has been observed to correlate with dispersal strength and social behaviour [29]. Thus, the study of shy-bold behaviour could potentially be very important to qualitatively and quantitatively predict not only population dispersal behaviour, but also invasion behaviour and disease spread due to the population structure. Future efforts should concentrate on adding a spatial dispersal component to the model to examine these implications more closely.
